# Transcriptome Analysis Reveals Molecular Signatures of Luteoloside Accumulation in Senescing Leaves of *Lonicera macranthoides*

**DOI:** 10.3390/ijms19041012

**Published:** 2018-03-28

**Authors:** Zexiong Chen, Guohua Liu, Ning Tang, Zhengguo Li

**Affiliations:** 1 Research Institute for Special Plants, Chongqing University of Arts and Sciences, Yongchuan 402160, China; chenzexiong1979@163.com (Z.C.); hongdou58771@sina.cn (G.L.); 2 Collaborative Innovation Center of Special Plant Industry in Chongqing, Chongqing 400000, China; 3 Chongqing Key Laboratory of Economic Plant Biotechnology, Chongqing 400000, China; 4 School of Life Sciences, Chongqing University, Chongqing 400030, China

**Keywords:** luteoloside biosynthesis, transcriptome analysis, *Lonicera macranthoides*, phenylpropanoid metabolism, transcription factors

## Abstract

*Lonicera macranthoides* is an important medicinal plant widely used in traditional Chinese medicine. Luteoloside is a critical bioactive compound in *L. macranthoides*. To date, the molecular mechanisms underlying luteoloside biosynthesis are still largely unknown. In this work, high performance liquid chromatography (HPLC) was employed to determine the luteoloside contents in leaves, stems, and flowers at different developmental stages. Results showed that senescing leaves can accumulate large amounts of luteoloside, extremely higher than that in young and semi-lignified leaves and other tissues. RNA-Seq analysis identified that twenty-four differentially expressed unigenes (DEGs) associated with luteoloside biosynthesis were significantly up-regulated in senescing leaves, which are positively correlated with luteoloside accumulation. These DEGs include *phenylalanine ammonia lyase 2*, *cinnamate 4-hydroxylase 2*, thirteen *4-coumarate-CoA ligases*, *chalcone synthase 2*, six *flavonoid 3′-monooxygenase* (*F3′H*) and two *flavone 7-O-β-glucosyltransferase* (*UFGT*) genes. Further analysis demonstrated that two *F3′Hs* (*CL11828.Contig1* and *CL11828.Contig2*) and two *UFGTs* (*Unigene2918* and *Unigene97915*) might play vital roles in luteoloside generation. Furthermore, several transcription factors (TFs) related to flavonoid biosynthesis including MYB, bHLH and WD40, were differentially expressed during leaf senescence. Among these TFs, *MYB12*, *MYB75*, *bHLH113* and *TTG1* were considered to be key factors involved in the regulation of luteoloside biosynthesis. These findings provide insights for elucidating the molecular signatures of luteoloside accumulation in *L. macranthoides*.

## 1. Introduction

*Lonicera macranthoides*, a member of the Caprifoliaceae family, is a medicinal plant primarily distributed in Southern China. It has been widely used as a critical raw material in traditional Chinese medicine for thousands of years, because it can effectively treat H1N1, respiratory syndrome, and hand-foot-and-mouth disease (China Pharmacopeia Commission, 2010). Furthermore, *L. macranthoides* extracts are utilized as important and indispensable ingredients in functional foods, beverages, wine and cosmetics [1,2]. A subset of secondary metabolites, including flavonoids, phenolic acid, volatile oil and saponins, is present in whole plants of *L. macranthoides* [3]. These components possess a wide range of pharmacological activities, such as antioxidant, anti-inflammatory, antibacterial, antiviral, antipyretic, liver protective and anticancer effects [4,5,6]. However, the quality and medicinal value of *L. macranthoides* remain controversial because of the distinctive accumulation patterns of bioactive compounds, especially flavones in flower buds. Luteolin and luteoloside are the main flavones in *Lonicera*. Previous studies demonstrated that the flavones are accumulated at high levels in *L. japonica*, which are approximately two fold higher than those in flower buds of *L. macranthoides* [7]. According to the China Pharmacopoeia, *L. macranthoides* was distinguished from *L. japonica* indicated by the report that luteoloside is less abundant in flowers of *L. macranthoides* than in those of *L. japonica* (China Pharmacopeia Commission, 2015). However, Yuan et al. [8] found no significant difference in luteoloside content between the flower buds of *L. japonica* and *L. macranthoides*. To date, there has been a longstanding controversy regarding the luteoloside accumulation in *L. macranthoides*. Hence, the knowledge of quantitative profiling for luteoloside is of major importance for characterizing molecular basis of luteoloside biosynthesis and has significant implications for health benefits of *L. macranthoides*.

Luteoloside is an important plant flavone [8], which exhibits diverse pharmacological activities for promoting nutrition and health [9]. Therefore, an increasing number of researchers attempted to increase biosynthesis and accumulation of luteoloside in *Lonicera* by using engineering strategies. Luteoloside is derived from the phenylpropanoid pathway, which can be divided into two stages [10]. First, phenylalanine is catalyzed to *trans*-cinnamic acid via phenylalanine ammonia-lyase (PAL, EC:4.3.1.24) and then to *p*-coumaric acid via *trans*-cinnamate 4-hydroxylase (C4H, EC:1.14.13.11). Subsequently, *p*-coumaroyl-CoA is generated via 4-coumarate-CoA ligase (4CL, EC:6.2.1.12), which is the precursor for a variety of important secondary metabolites [11]. Yuan et al. [8] observed that the expression of *PAL1*, *C4H2* and *4CL2* positively correlates with the content of luteolin, the substrate of luteoloside biosynthesis, in the leaves and flower buds of *L. japonica*. These findings are consistent with the reports showing a positive correlation was found between the enzyme activities of PAL, C4H and 4CL and the accumulation of luteoloside [1]. The above results indicate the essential roles of these genes in luteoloside biosynthesis in *L. japonica*. Chalcone synthase (CHS, EC:2.3.1.74) and chalcone isomerase (CHI, EC:5.5.1.6) are the key regulatory enzymes required for the conversion of *p*-coumaroyl-CoA to naringenin [12].Naringenin serves as an important branch point for luteoloside biosynthesis, which is catalyzed to either apigenin or eriodictyol through flavones synthase (FNS, EC:1.14.11.23) or flavonoid 3′-hydroxylase (F3′H, EC:1.14.13.21), respectively. The two flavones are converted to luteolin via F3′H and FNS, respectively. Luteoloside is further synthesized via UDP-glucose flavone 7-*O*-β-glucosyltransferase (UF7GT, EC:2.4.1.81) [13]. CHI exhibits a close correlation with variations in luteoloside content at different flowering stages in *L. japonica* [1,8]. Other genes encoding CHS, FNSII, F3′H and UFGT were identified by RNA-Seq to be possibly associatedwith luteoloside biosynthesis in *L. japonica* [10,14]. Very recently, Wu et al. [7] reported a FNSII gene, *LmFNSII-1.1*, might be the key gene involved in flavone accumulation in the buds of *L. macranthoides*. Despite recent advances, the publications concerning the molecular evidence for a relationship between luteoloside abundance and organ development of *L. macranthoides* are limited. It remains a mystery that which enzymes or genes are responsible for luteoloside accumulation in *L. macranthoides*.

Flavonoid biosynthesis involves a multitude of regulatory proteins. Some transcription factors (TFs), such as R2R3-MYB, bHLH and WD40, can regulate the genes encoding biosynthetic enzymes by binding their promoters. To date, MYBs controlling flavonoid biosynthesis have been identified in many plant species. *MYB11/12/111* in *Arabidopsis* [15,16], *VvMYBF1* in *Vitis vinifera* [17] and *EsMYBF1* in *Epimedium sagittatum* [18] were characterized to activate flavonol accumulation. Several MYBs, including *MdMYBA* [19], *MdMYB1* [20] and *MdMYB10* [21] in apple, *VvMYBA1* and *VvMYBA2* in grape [22,23], and *PyMYB10* in pears [24] control anthocyanin biosynthesis positively. Aside from the activators, several repressor MYBs are also isolated, including *AtMYBL2* [25], *AtMYB4*, *AtMYB60* [26,27], *AmMYB308* [28] and *FaMYB1* [29]. Furthermore, several bHLH and WD40 proteins, such as TT8 and TTG1, act as positive regulators for flavonoid biosynthesis [30]. Although more TF members of MYB-bHLH-WD40 related to flavonoid biosynthesis are characterized, scare reports on the transcriptional regulation of flavonoid biosynthesis in *Lonicera* are available [31]. Thus, identifying the TFs involved in the regulation of luteoloside accumulation is essential for elucidating the molecular features of luteoloside biosynthesis.

In the present study, luteoloside in different tissues and developmental stages was determined using high performance liquid chromatography (HPLC) and transcriptome-wide sequencing in *L. macranthoides* was performed using the Illumina HiSeq™ 2000 platform to explore the key genes encoding biosynthetic enzymes and regulator proteins associated with the luteoloside biosynthesis in *L. macranthoides*. These results provide genomic resources for future enhancement of luteoloside production by genetic strategies in *L. macranthoides*.

## 2. Results

### 2.1. Luteoloside Contents in Different Tissues and Developmental Stages of L. macranthoides

**Figure 1:**
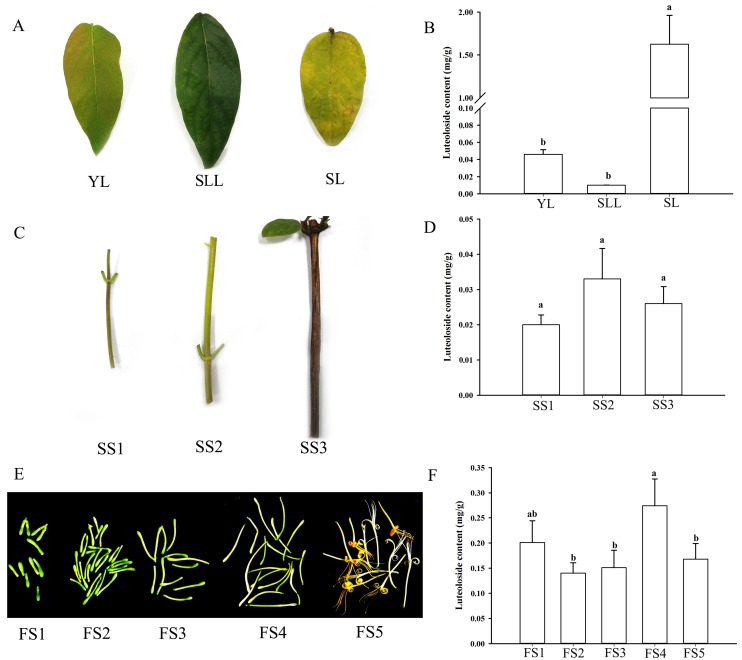
The luteoloside contents in different tissues at different developmental stages in *Lonicera macranthoides*. (**A**). Leaves at three developmental stages. YL, SLL and SL indicate young leaves, semi-lignified leaves and senescing leaves, respectively. (**B**). Luteoloside contents in leaves were determined by HPLC. (**C**). Stems at three developmental stages. SS1, SS2 and SS3 indicate young stems, semi-lignified stems and senescing stems, respectively. (**D**). Luteoloside contents in stems were determined by HPLC. (**E**). Flowers at five stages of development, including green flower buds in length of 10 mm (FS1) and 20–30 mm (FS2), white flower buds in length of 30–40 mm (FS3), white flower (FS4) and yellow flower (FS5). (**F**). Luteoloside contents in flowers were determined by HPLC. Three biological replications were performed for each examination. Values are means ± SD (*n* = 3). Duncan’s multiple range test was used to analyze the significance and the different lower-case letters (e.g., a and b) indicate significant (*p* < 0.05) differences between samples.

Luteoloside contents in the leaves, stems and flowers were quantified by HPLC (Figure S1). The contents differed significantly among the three tissues and different developmental stages (Figure 1). Senescing leaves (SL) can accumulate very large amounts of luteoloside (up to 1.625 mg/g DW), which was approximately 35- and 160-fold higher than that in young leaves (YL) and semi-lignified leaves, respectively (Figure 1A,B). There was no obvious change in luteoloside accumulation among the various developmental stages of stems (Figure 1C,D). Luteoloside contents fluctuated throughout the flower development and the highest luteoloside level in flowers was found in white flower, followed by green flower buds in length of 10 mm (Figure 1E,F). For all tissues examined, flowers displayed remarkably higher luteoloside contents than that in leaves and stems of various developmental stages except for senescing leaves.

### 2.2. RNA-Seq Analysis

To further explore the candidate genes involved in luteoloside biosynthesis of *L. macranthoides*, two libraries including SL and YL were established for RNA-Seq analysis. The raw data generated by sequencing for each library ranged from 82.4 to 87.5 million reads. After filtering, a total of 76.8 to 82 million clean reads were obtained, accounting for 93% of the raw reads, with an average GC % of 44.19% for all clean reads being (Supplementary Table S1).

These clean reads were assembled into 260,079 and 173,214 contigs for SL and YL libraries, respectively. After processing by using Trinity and TGICL software, the contigs of SL were further incorporated into 183,667 unigenes with an average length of 903 bps and an N50 length of 1501 bps. Moreover, a total of 122,824 unigenes were obtained in YL library, with an average length of 1131 bp and an N50 value of 1859 bps (Table 1). The length distribution for all unigenes in *L. macranthoides* is shown in Figure S2. The numbers of unigenes with sequence length longer than 500 bp, 1000 bp, 2000 bp and 3000 bp accounted for 70.05% (111,272), 41.3% (65,614), 16.8% (26,673) and 5.7% (9053) of the total unigenes, respectively (Figure S2). The assembled data for *L. macranthoides* in this study was improved compared to our earlier published transcriptome assemblies, with remarkable increases in overall unigene length and N50 value [11].

The unigene sequences were subjected to BLASTx for further annotation, with a threshold of 10^−5^. A total of 111,811 unigenes (70.4%) showed significant similarity to known proteins. Among them, 101,618, 68,308, 68,713, 67,523, 55,312 and 56,734 unigenes can be annotated in the NCBI nonredundant (Nr), nucleotide sequence (Nt), Swiss-Prot, Kyoto Encyclopedia of Genes and Genomes (KEGG), Clusters of Orthologous Groups of proteins (COG), and GO databases, respectively (Supplementary Table S2). Similarity distribution analysis of the top-hits in the Nr database indicated that 49% of the unigenes showed more than 60% similarity (Figure S3A). Species distribution analysis demonstrated that 42.6% of the unigenes showed close homology with seven plant species, including *V. vinifera*, *‘Chlorella vulgaris’ C-169*, *Theobroma cacao*, *Solanum tuberosum*, *Amygdalus persica*, *Hordeum sativum* and *Populus trichocarpa*, of which, 17.8% of the annotated sequences were assigned to *V. vinifera* (Figure S3B).

### 2.3. Differentially Expressed Unigenes (DEGs) in Senescing and Young Leaves

To improve the accuracy of the RNA-Seq data, two independent biological replicates were performed for each sample in this study. The Pearson and Spearman correlation coefficients were calculated to investigate the correlation of the gene expression data among the biological replicates and visualized with a color-coded diagram. The Pearson correlation coefficients were 0.99 and 0.83 between the two libraries of SL and YL, respectively. And the Spearman correlation coefficients were 0.79 and 0.95 (Figure S4A,B). These results indicate strong correlation between variables and reliability of the RNA-Seq data.

**Figure 2:**
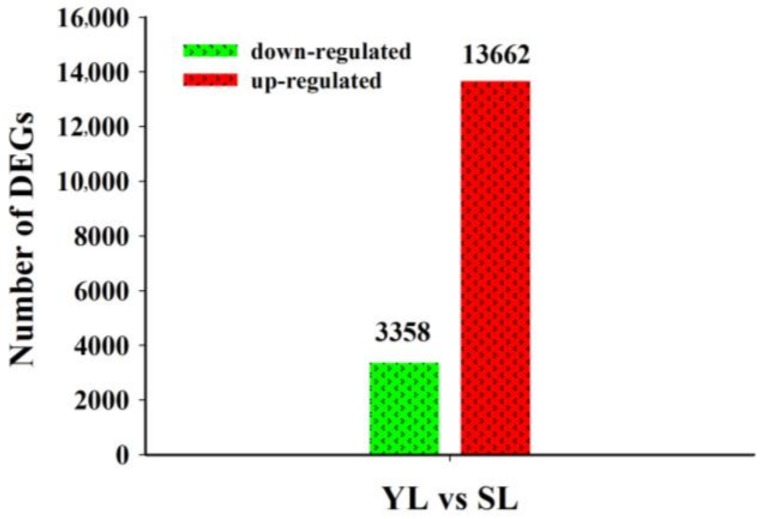
Number of differentially expressed unigenes during leaf senescence identified by RNA-Seq data in *Lonicera macranthoides*. Differentially expressed unigenes (DEGs) between young and senescing leaves were illustrated by bar chart. Red and green bars represent the up-regulated and down-regulated unigenes in senescing leaves (SL) compared with those in young leaves (YL), respectively in *Lonicera macranthoides*.

The DEGs between senescing leaves and young leaves were identified (Supplementary Table S3), and the distribution of log-fold changes was visualized in the volcano plot (Figure S4C). With respect to young leaves, a total of 17,020 unigenes were differentially expressed in senescing leaves, of which 13,662 unigenes were up-regulated and 3,358 unigenes were down-regulated (Figure 2).

### 2.4. GO- and KEGG-Based Functional Classification of DEGs

**Figure 3:**
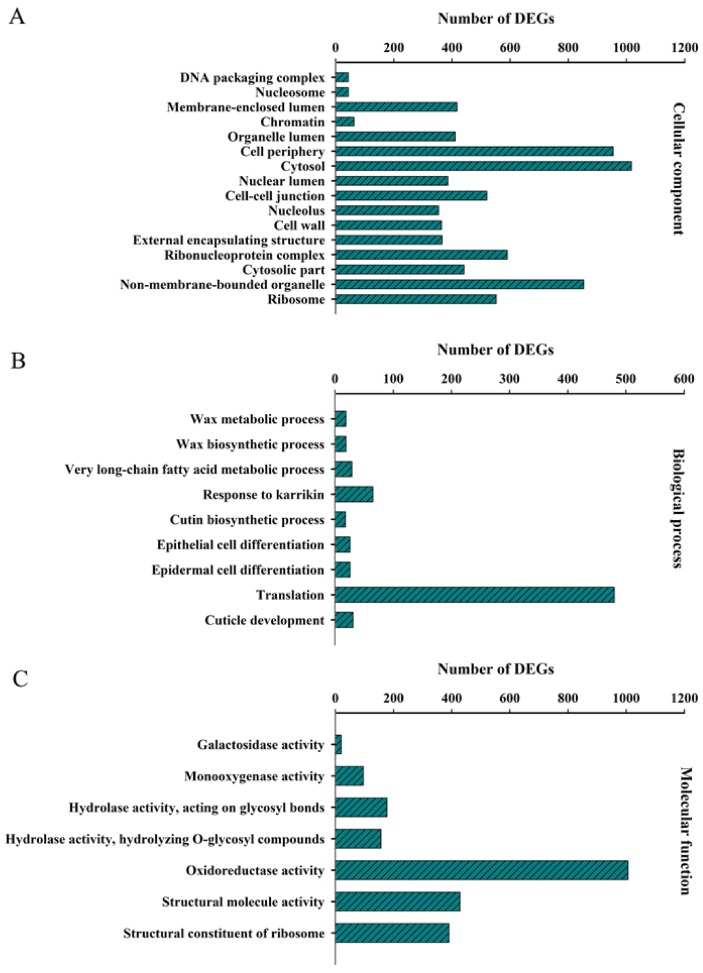
Gene Ontology enrichment analysis of DEGs during leaf senescence in *Lonicera macranthoides*. Significantly enriched gene ontology (GO) terms (FDR < 0.05) for DEGs between young and senescing leaves based on cellular component (**A**), molecular function (**B**) and biological process (**C**) were identified using the Blast2GO program. The left *y*-axis indicates the enrichment GO terms and the top *x*-axis indicates the number of DEGs in each category.

To gain an insight into the functional characterization of DEGs, GO-based classification was performed by using the Blast2GO program v 3.0 [32]. Significantly enriched GO categories were identified (*p* < 0.05), 16 for cellular component (Figure 3A), 9 for biological process (Figure 3B), and 7 for molecular function (Figure 3C). Several biological processes, such as cuticle development, epidermal and epithelial cell differentiation, long-chain fatty acid metabolic process and wax biosynthetic process, were differentially regulated at various developmental stages of leaves (Figure 3B). Moreover, for the molecular function category, GO terms corresponding to oxidoreductase activity, hydrolase activity and monooxygenase activity were remarkably enriched (Figure 3C). This result indicates the potential roles of these GO terms in luteoloside biosynthesis.

To better understand the gene function, we assigned 10,216 of the DEGs to one of 128 KEGG terms by using the KEGG database. Significantly altered biological pathways were identified during leaf senescence (Q value ≤ 0.05). Several metabolic pathways, including biosynthesis of secondary metabolites, phenylpropanoid biosynthesis, lipid and fatty acid metabolism, starch and sucrose metabolism, tryptophan metabolism, cutin and wax biosynthesis, and flavone and flavonol biosynthesis displayed outstanding enrichments (Figure S5). This finding indicates a significant difference in primary and second metabolism between young and senescing leaves in *L. macranthoides*. A total of 339 and 67 DEGs were assigned to phenylpropanoid metabolic and flavone/flavonol biosynthetic pathways, respectively (Figure S5), by which generate precursors for luteoloside biosynthesis.

### 2.5. Identification of Candidate Genes Associated with Luteoloside Biosynthetic Pathways

**Figure 4:**
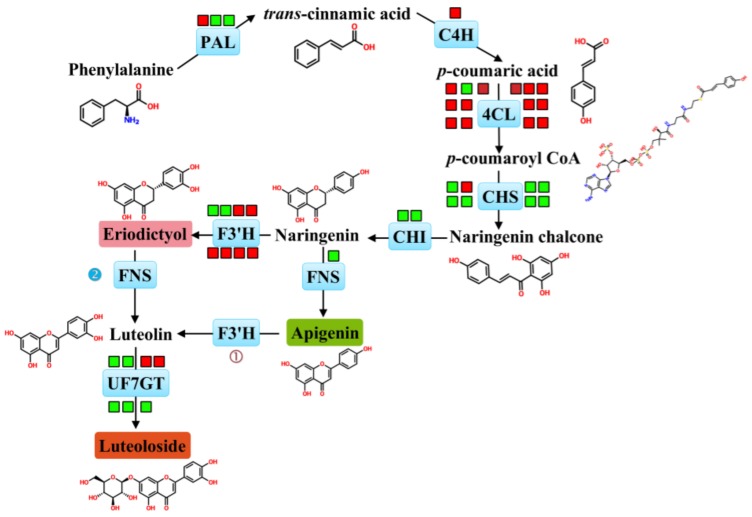
Transcriptomic mapping of genes associated with luteoloside biosynthesis in *Lonicera macranthoides*. Proposed pathways for luteoloside biosynthesis in *Lonicera macranthoides* were illustrated by RNA-Seq analysis. Luteolin, the precursor of luteoloside, is biosynthesized from the general flavonoid precursor: naringenin. Circle 1(①) indicates that luteolin is biosynthesized directly from apigenin catalyzed by F3’H. Circle 2(②) indicates that luteolin is generated directly from eriodictyol catalyzed by FNS. Expression profile for each gene was shown in colored blocks and each blocks represented the expression changes (represented by Log2Ratio) in senescing leaves with respect to young leaves. Red colors/green colors correspond to up-/down-regulation of these genes and Log2Ratio ≥ 1 is considered statistically significant. Details were showed in Table 2. Abbreviations: PAL, phenylalanine ammonia lyase; C4H, cinnamate 4-hydroxylase; 4CL, 4-hydroxycinnamoyl CoA ligase/4-coumarate-CoA ligase; CHS, chalcone synthase; CHI, chalcone isomerase; FNS, flavone synthase; F3H, flavonoid 3′-monooxygenase/flavonoid 3′-hydroxylase; UF7GT, flavone 7-*O*-β-glucosyltransferase.

**Figure 5:**
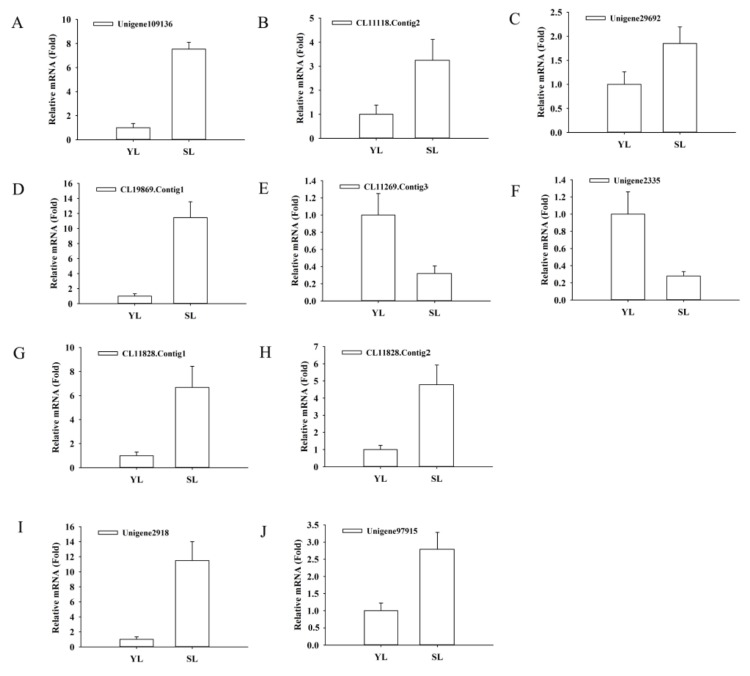
Expression patterns of selected unigenes related to luteoloside biosynthesis identified by RNA-Seq were validated by qRT-PCR. Expressions of unigenes located upstream of luteoloside metabolic pathway (**A**) *Unigene109136* (*PAL*), (**B**) *CL11118.Contig2* (*C4H*), (**C**) *Unigene29692*(*4CL*) and downstream of luteoloside metabolic pathway (**D**) *CL11269.Contig3*(*CHI*), (**E**) *CL19869.Contig1*(*CHS*), (**F**) *Unigene2335*(*FNSII*), (**G**) *CL11828.Contig1* and (**H**) *CL11828.Contig2*(*F3’H*), (**I**) *Unigene2918* and (**J**) *Unigene97915*(*UFGT*) in young and senescing leaves were analyzed by qRT-PCR. Relative expression levels were determined based on the reference young leaves set to 1. Values are means ± SD of three biological repetitions (*n* = 3). Two technical replicates for each biological replicate were performed in each qRT-PCR experiments.

To elucidate the key genes for luteoloside biosynthesis, DEGs involved in phenylpropanoid pathway were screened, including three *PAL*, one *C4H*, fourteen *4CL*, eight *CHS*, two *CHI*, one *FNS*, eight *F3′H* and seven *UFGT* genes (Figure 4). *PAL2* (*Unigene109136*), *C4H2* (*CL11118.Contig2*) and the majority of *4CL* genes (13 out of 14) were significantly upregulated in senescing leaves compared with young leaves. We also found that *CHS2* (*CL19869.Contig1*), six *F3′H* (*CL11828.Contig1*, *CL11828.Contig2*, *CL7653.Contig1*, *Unigene102655*, *Unigene65437* and *Unigene76746*), two *UFGT* (*Unigene2918* and *Unigene97915*) genes displayed dramatic increases in transcript levels during leaf senescence. Meanwhile, *CHI* and *FNS* paralogous genes appeared remarkable down-regulation in senescing leaves, compared with those in young leaves (Table 2). The mRNA levels of ten unigenes possibly involved in luteoloside biosynthesis were validated by qRT-PCR, which displayed similar expression patterns to the results obtained by RNA-Seq (Figure 5A–J).These DEGs, especially those functioning downstream in the metabolic pathway, may provide valuable clues to illustrate the luteoloside biosynthetic pathway. The high expression levels of *CHS2*, *F3′H* and *UFGT* genes in senescing leaves were consistent with the large amounts of luteoloside, indicating their fundamental involvement in luteoloside biosynthesis.

To further characterize the molecular properties of the *F3′H* and *UFGT* genes, phylogenetic analyses of protein sequences were performed. Two *F3′H* genes, namely *CL11828.Contig1* and *CL11828.Contig2*, were grouped into the same clade as the *F3′H* genes in *Sesamum indicum*, *Solanum lycopersicum*, *Ziziphus jujube* and *Nicotiana tabacum* and also shared high sequence homology to the *F3′H* genes in *Arabidopsis lyrata* and *Oryza sativa*. This result suggests the similar functions of these *F3′H* genes. However, the four remaining *F3′H* genes showed relatively lower levels of similarity to those genes in other species (Figure S6A). Two UFGTs were classified into the same subcluster with UDP-glucose flavonoid 3-*O*-glucosyltransferase (UF3GTs) in several species and also displayed amino acid sequence similarities with UF7GTs in *Arabidopsis* and *Scutellaria* (Figure S6B).

### 2.6. Identification of DEGs Involved in MYB, bHLH and WD40 Transcription Factors

**Figure 6:**
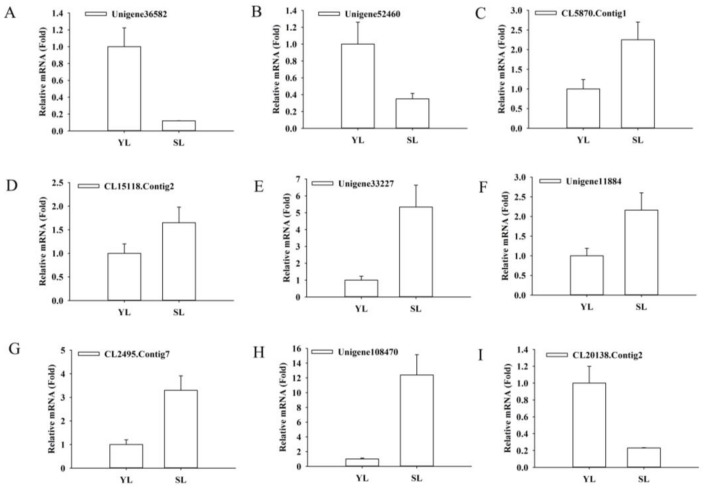
Expression patterns of selected unigenes related to transcription factors identified by RNA-Seq were validated by qRT-PCR. Expressions of unigenes, homologs to *AtMYB12* (**A**) *Unigene36582* and (**B**) *Unigene52460*, homologs to *VvMYB75* (**C**) *CL5870.Contig1*, homologs to *VvMYB1R1* (**D**) *CL15118.Contig2*, homologs to *AtbHLH113* (**E**) *Unigene33227* and (**F**) *Unigene11884*, homologs to *AtbHLH78* (**G**) *CL2495.Contig7*, homologs to *TTG1* (**H**) *Unigene108470* and homologs to *AtMYB5* (**I**) *CL20138.Contig2* in young and senescing leaves were analyzed by qRT-PCR. Relative expression levels were determined based on the reference young leaves set to 1. Values are means ± SD of three biological repetitions (*n* = 3). Two technical replicates for each biological replicate were performed in each qRT-PCR experiments.

MYB, bHLH, and WD40 have been previously identified to regulate flavonoids biosynthesis [30]. RNA-seq data showed that thirty MYB, thirty-one bHLH and eighty-six WD40 TFs in senescing leaves displayed differential expression with respect to young leaves (Table 3 and Supplementary Table S4). In contrast to young leaves, approximately half of the differentially expressed *MYBs* (16 out of 30) underwent significant down-regulation in senescing leaves, such as those orthologous to *TT2*, *AtMYB12*, *PsMYB1*, *SlMYB46*, *VvMYB44*, and *VvMYB306*. Meanwhile, the remaining differentially expressed MYBs, including those homologous to *AtMYB48*, *VvMYB1R1*, *VvMYB75*, *AmMYB305*, *EsMYB13*, and *GhMYB9*, showed dramatically increased transcripts in senescing leaves (Table 3). Moreover, out of the 30 bHLHs, 14 bHLHs, which include *bHLH25*, *bHLH49*, *bHLH66*, *bHLH78*, *bHLH113*, *bHLH123* and *bHLH143*, were strongly up-regulated during leaf senescence (Table 3). In addition, among the DEGs involved in WD40 repeat-containing proteins, one transparent testa glabrous1 (TTG1) ortholog was remarkably upregulated in senescing leaves compared with that in young leaves (Supplementary Table S4). This gene contained five WD repeats and showed 37% amino acid identity with TTG1 in *Arabidopsis* (XP_020877597), 38% identity with TTG in *Medicago* (XP_003624554) and 37% identity with TTG1 in *Malus* (AHM88209). QRT-PCR analysis also showed that the transcripts of several unigenes, including those homologous to MYB, bHLH and TTG1, were in dramatic regulation during leaf senescence (Figure 6A–I).

## 3. Discussion

Luteoloside belongs to a group of natural flavonoids isolated from *Lonicera*, that exerts human health benefits, including antiallergic, free radical scavenging and antioxidant, antihyperglycemic and anticholestatic activities [9]. Luteoloside accumulation is a precisely regulated process that varies considerably depending on plant species, developmental stages and different tissues. To date, fingerprint analysis of luteoloside has been carried out in *Lonicera*. The amounts of luteoloside are remarkably lower in flower buds of *L. hypoglauca* than inthose of *L. japonica*, whereas no obvious change is observed between the flower buds of *L. japonica* and *L. macranthoides* [8]. However, according to the description in the Chinese pharmacopoeia 2010 and the view reported by Wu et al. [7], luteolin and luteoloside are in lower abundance in flower buds of *L. macranthoides* than in those of *L. japonica* [33]. In the present study, luteoloside accumulation initially increased from stage 2 to stage 4 and then decreased in stage 5 during flower development in *L. macranthoides* (Figure 1F), which is in accordance with the results of previous reports in *L. japonica* [1,7]. Moreover, in contrast to the findings of Yuan et al. in *L. japonica* [8], our results revealed that luteoloside displayed significantly higher level in flowers than that in stems and leaves at different growth stages except for senescing leaves (Figure 1). In general, luteoloside is relatively low in *L. macranthoides* (only 0.01 to 0.2 mg/g). Our study demonstrated that luteoloside was accumulated at extremely higher level in senescing leaves of *L. macranthoides* (Figure 1B). This finding provides us with the view of significant medical value of the senescing leaves and new insights into the molecular basis underlying luteoloside biosynthesis.

### 3.1. Molecular Features Underlying Abundant Accumulation of Luteoloside during Leaf Senescence in L. macranthoides

Transcriptome analysis in leaves at different developmental stages was aimed at elucidating molecular mechanism underlying high accumulation of luteoloside in senescing leaves of *L. macranthoides*. Several genes encoding PAL, C4H and 4CL displayed higher transcript levels in senescing leaves (Figure 4), which might contribute to luteoloside accumulation and thus were considered to be critical members associated with luteoloside generation. These findings are in accordance with the previous observations that the activities and expressions of PAL, C4H and 4CL positively correlate with luteoloside biosynthesis [1,8]. However, the three enzymes are located upstream of the luteoloside metabolic pathway and as common enzymes regulating biosynthesis of various secondary metabolites, such as lignin [34] and flavonoids [35]. Therefore, other genes enabling irreversible commitments to luteoloside biosynthetic pathways need to be further investigated. *LjCHS1* and *LjCHI2* were proposed to be the important genes participating in luteolin biosynthesis [8], whereas the transcripts of two *CHI* genes exhibited negative correlation with luteoloside levels in our study (Figure 4), suggesting that the regulation of luteoloside accumulation differs among plant species.

Three enzymes including F3′H, FNS and UFGT, are proved to play crucial roles in luteoloside metabolic pathway [7,10,14,36]. In the present study, the enhanced expression of two *F3′H* homologs (*CL11828.Contig1* and *CL11828.Contig2*) coincided precisely with the high level of luteoloside in senescing leaves (Table 2), indicating that they are potential candidates modulating the biosynthesis of luteolin and luteoloside. *CYP75B3*, a *F3′H* gene in *Oryza sativa*, exhibits similar preference for naringenin and apigenin, in nearly catalytic efficiencies for these substrates [36]. The two *F3′H* genes showed close homology with CYP75B3 (Figure S6A), suggesting that these genes were not altered to exhibit flavonoid-3-hydroxylation activity and might facilitate the biosynthesis of eriodictyol or luteolin from naringenin or apigenin, respectively. FNS is considered as the critical enzyme in the two routes of luteoloside metabolic pathway (Figure 4), which is implied by the accumulation of luteolin-7-*O*-glucoside (luteoloside) and apigenin-7-*O*-glucoside in *FNSII*-overexpression transgenic lines in *Lonicera* [7]. Wu et al. [7] also demonstrated that FNSII in *L. japonica* (*LjFNSII-1.1*) exhibits an apparently higher catalytic activity (approximate 4-fold) than that in *L. macranthoides* (*LmFNSII-1.1*). They concluded that the less abundance of flavones in flowers of *L. macranthoides* compared with that in *L. japonica* might be attributed to the weak catalytic efficiency of LmFNSII. However, in our dataset, a FNSII gene (*Unigene2335*) exhibiting 99% identity with *LmFNSII-1.1* (deletion of a leucine at position 22 in *LmFNSII-1.1*) displayed lower transcript abundance in senescing leaves than in young leaves (Table 2). Thus, it seems not to be responsible for luteoloside generation. In addition, high levels of luteoloside in senescing leaves might result from the highly-expressed UFGTs (*Unigene2918* and *Unigene97915*), homologs of *AtUF7GT* and *SbUF7GT* (Figure S6B), which can maximize the luteolin conversion. Hence, the extremely higher amounts of luteoloside in senescing leaves might be attributed to significantly higher transcript levels of *F3′H* in senescing leaves compared to that in young leaves. The increase in *F3′H* expression might provide sufficient eriodictyol to FNSII, and then large amounts of luteolin would accumulatedue to the relatively high background expression of FNSII (Table 2). Given that the FNSII enzymes prefer eriodictyol as a substrate over naringenin in *Lonicera* [7], the role of FNSII in luteolin generation from the other routes (naringenin-apigenin-luteolin) could be neglected. On the basis of our results and the findings in the literature described above, we hypothesized that luteoloside is preferentially biosynthesized via the following alternative route: naringenin is hydroxylated by F3′H (*CL11828.Contig1* and *CL11828.Contig2*) to produce eriodictyol and then FNSII converts eriodictyol to luteolin, which can be metabolized to luteoloside via UFGTs. Therefore, although the transcript levels of *FNSII* in young leaves were approximately 7-fold higher than that in senescing leaves (Table 2), lower levels of luteoloside accumulation were observed due to the lower expressions of *F3′Hs* in young leaves than that in senescing leaves (Figure 1B).

### 3.2. Transcription Factors as Potential Regulators of Luteoloside Accumulation in Leaves of L. macranthoides

Flavonoid biosynthesis is transcriptionally regulated by a multitude of transcription factors, of which R2R3-MYB, bHLH and WD40 repeat proteins are proven to be vital [37]. Numerous studies have demonstrated that MYB12/11/111 can modulate flavonol synthesis by activating the early biosynthetic genes including *PAL*, *C4H*, *CHS*, *CHI* and *F3′H* [16,17,38,39,40,41]. A recent study in *E. sagittatum* showed that *EsMYBF1*, a homolog of *AtMYB12* and *VvMYBF1*, functions as an activator regulating the flavonol synthesis. Ectopic expression of *EsMYBF1* resulted in the enhanced accumulation of kaempferol and quercetin via upregulating the expression of *CHS*, *CHI*, *F3H* and *FLS* genes but a decline in the content of anthocyanin via downregulating the transcripts of *DFR* and *ANS* genes, suggesting that *EsMYBF1* is a flavonol-specific regulator [18]. Moreover, our results indicated that two putative *MYB12* genes in *L. macranthoides* (*Unigene36582* and *Unigene52460*), which are homologs of *AtMYB12*, were more abundantly expressed in young leaves (Table 3), in which the phenylpropanoid products, chlorogenic acid and rutin, were largely accumulated (unpublished data) but extremely low content of luteoloside was observed (Figure 1B). These findings suggest that the two *MYB12* homologs might regulate the phenylpropanoid and flavonoid biosynthetic pathways in *L. macranthoides*. The disparate accumulation patterns of the related main metabolites and derivatives upon both up- and down-regulation of *MYB12* might be preferentially ascribed to a flux shift in the metabolic pathway rather than a direct outcome of transcriptional activation or repression in leaves, which is manifested by the reports that overexpression of *AtMYB12* in tomato activates the caffeoylquinic acid biosynthetic pathway, while down-regulation of *SlMYB12* also leads to the accumulation of caffeic acid derivatives [42]. Moreover, several novel MYBs were observed to correlate with the accumulation pattern of luteoloside during leaf senescence, in particular the positive-correlated genes, such as those homologs to *AtMYB48*, *VvMYB1R1*, *VvMYB75*, *AmMYB305*, *EsMYB13* and *GhMYB9* (Table 3), indicating their involvement in the regulation of luteoloside biosynthesis in *L. macranthoides* leaves.

bHLH, the key component involved in the regulation of flavonoid biosynthesis, was observed to activate late biosynthetic genes (i.e., *F3′H*, *ANR*, *DFR*, and *UFGT*) through formation of highly dynamic MYB/bHLH/WD40 (MBW) complexes [43,44,45]. In Arabidopsis, three bHLH activators including TRANSPARENT TESTA 8(TT8), enhancer of glabra3 (EGL3), glabra3 (GL3), were reported to act in the transcriptional regulation of anthocyanin and proanthocyanidins (PAs) production via interacting directly or indirectly with Mybs [30,46]. Other bHLH proteins, such as MdbHLH3 in apple and BobHLH1 in cauliflower, interact with MdMYB10 and BoMYB2 respectively, were observed to confer anthocyanin accumulation [21,47]. In the current study, a subset of bHLHs, such as two *AtbHLH113* homologs (*CL3655.Contig4* and *Unigene11884*) and four *AtbHLH78* homologs, displayed higher mRNA levels in senescing leaves than that in young leaves, which is positively correlated with the accumulation of luteoloside, suggesting their involvements in the regulation of luteoloside generation. This result is in accordance with the findings in *Arabidopsis* that AtbHLH113 is predicted to interact with PAP1/MYB75 modulating anthocyanin biosynthesis [48].

WD40 repeat protein is another pivotal factor of MBW complexes [46]. In this study, a TTG1 ortholog (*Unigene108470*) (Supplementary Table S4) in *L. macranthoides* was isolated and displayed a positive correlation with luteoloside production. In terms of the roles of TTG1 in regulating flavonoid biosynthesis, we considered that *Unigene108470* might be the candidate factor in controlling luteoloside biosynthesis. The transcription factors, particularly R2R3 MYB TFs, may activate distinct sets of structural genes of flavonoid biosynthesis [49]. In our dataset, several novel candidate genes participating in the transcriptional regulation of flavonoids biosynthesis in *L. macranthoides* were identified. Further work will be required to determine whether these genes significantly induce or limit luteoloside biosynthesis in plants and if so, their molecular mechanisms have to be explored via genetic and biochemical approaches in vitro and in vivo.

## 4. Materials and Methods

### 4.1. Plant Materials

*Lonicera macranthoides* Hand-Mazz (cv. Yu Lei 1#) used in the experiments was planted in a greenhouse of Chongqing University of Arts and Sciences (Chongqing, China). Plant tissues including leaves, stems and flowers at different developmental stages were collected from 5-year-old seedlings. Leaves and stems at three developmental stages including young leaves and stems, semi-lignified leaves and stems, senescing leaves and stems were obtained and illustrated in Figure 1A, and C. Flowers at five developmental stages were harvest in June. These stages are illustrated in Figure 1E. Stage 1 and stage 2 flowers are defined by green flower buds with length of 10 mm and 20–30 mm, respectively. Stage 3flowers are defined by white flower buds with length of 30–40 mm, while stage 4 and stage 5 flowers are defined by white flowers and yellow flowers, respectively. All the samples used for HPLC and RNA-Seq analysis were collected from 18 individual plants and tissues from 6 plants were pooled and set as one replicate. Three biological replicates were performed in each experiment (approximately 25 leaves, 25 stems, and 40 flowers in each replicate). All collected samples were placed in 50mL Falcon tubes, frozen in liquid nitrogen and then stored at −80 °C until further use.

### 4.2. Determination of Luteoloside Contents by High Performance Liquid Chromatography (HPLC)

Tissue samples were subjected to lyophilization and homogenization with a grinding miller. The powder was then passed through a 40-mesh (420 μm) standard sieve before extraction. A total of 0.25 g homogenized samples was extracted with 20 mL of ethanol (70%, *v*/*v*) by ultrasonication for 30 min. The extracts were cooled to room temperature and centrifuged at 4000 rpm for 10 min. Afterward, the supernatant was filtered through a 0.22 µM microfiltration membrane for luteoloside analysis by HPLC.

HPLC was performed on a Shimadzu LC-20A HPLC analyzer (Shimadzu Corporation, Kyoto, Japan), equipped with a LC-20AT pump, a SIL-20A auto sampler, a CBM-20A system controller, a SPD-M20A diode array detector (DAD)and a CTO-20A column oven. Separation was carried out on a Shimadzu Shim-Pack VP-ODS C18 column (5 μm, 250 × 4.6 mm) using gradient elution. Mobile phase A was 2% formic acid in deionized water, while phase B was 2% formic acid in methanol (80:20, *v*/*v*). A 15% B linear gradient was conducted from 0.01 min to 8.00 min, followed by a 15~50% B linear gradient from 8.01 min to 25.00 min, and finally a 50% B linear gradient from 25.01 min to 40.00 min, at a flow rate of 0.7 mL/min. The column and the detector were operated at 35 °C. A volume of 20 μL supernatant was injected and the detection wavelength was monitored at 360 nm. Luteoloside standard (>98%) was purchased from SIGMA (Sigma-Aldrich, St. Louis, MO, USA). The luteoloside contents were analyzed in triplicate and calculated based on peak area measurements. Statistical significance was performed with SPSS using Duncan’s new multiple range test.

### 4.3. RNA Extraction and Illumina Sequencing

Total RNA from young and senescing leaves was isolated and purified using QIAGEN RNeasy Plant Mini kit and RNase-free DNase set (QIAGEN, Hilden, Germany) according to manufacturer’s instruction. RNA-Seq was performed at Hangzhou 1GENE Technology Co., Ltd. (Hangzhou, China). Illumina TruSeq™ RNA Sample Prep Kit (Cat# RS-122-2001) (Illumina, San Diego, CA, USA) was employed to construct cDNA library according to the manufacturer’s protocol. Briefly, Poly(A) mRNA was enriched from 5 µg total RNA using oligo (dT) magnetic beads and fragmented in a thermomixer. The short fragments were used as templates to synthesize first- and second-strand cDNAs. Afterward, cDNA was end-repaired, A-tailed and ligated with Illumina-specific adaptors. Finally, the fragments were size selected and PCR amplified to generate the cDNA library. RNA-sequencing was performed using the Illumina HiSeq™ 2000 platform. Each sample was analyzed in two biological replicates in this RNA-Seq experiment.

### 4.4. De Novo Transcriptome Assembly and Functional Annotation

The raw reads were filtered by removing adaptors and low-quality reads. After that, the clean reads were generated. Trinity (version 2.0.6) (http://trinityrnaseq.github.io/) and TGICL software (version r2013-04-11) (https://sourceforge.net/projects/tgicl/) were employed to assemble our clean data. The clean reads from the SL and YL libraries were first processed independently. To obtain complete reference sequences, the clean reads from all the samples were mixed and assembled again. To assess the quality of de novo assembly, the length distribution of assembled contigs and unigenes were collected. To obtain functional annotation of a given unigene, the sequence was aligned against protein sequence database entries including those in the Nr, Swiss-Prot, KEGGand COG databases using BLASTx (E-value < 0.00001). The unigenes were also aligned against the Nt database using BLASTn and we declared sequences similar if the corresponding E-value for the alignment was less than 10^−5^.

### 4.5. Screening of Differentially Expressed Unigenes

Gene expression was calculated using the number of reads aligned to a single gene and the total number of reads aligned to reference sequences in the reads per kb per million reads (RPKM) method [50]. R package DEGseq was employed to identify DEGs with random sampling model [51]. A *p*-value can denote its expression difference between the two libraries, while false discovery rates (FDRs) were used to determine the threshold of *p* value. We set “FDR < 0.001 and the absolute value of log2Ratio > 1” as the threshold to judge the significance of gene expression difference according to described method in the literature [52].

### 4.6. Validation of RNA-Seq Data by Quantitative Real-Time PCR (qRT-PCR)

To validate the accuracy of the gene expression levels of DEGs obtained from the RNA-Seq analysis, 19 genes possibly associated with luteoloside synthesis were randomly selected and subjected to qPCR detection. Gene-specific primers for the selected genes were designed using an online primer design software (https://www.genscript.com/ssl-bin/app/primer) (Supplementary Table S5) and a melting curve analysis was used to confirm specificity. qRT-PCR was performed using a Fast SYBR Mixture (CWBIO, Beijing, China) on a Bio-Rad CFX connect real-time PCR detection system using of 95 °C incubation for 10 min, then 40 cycles of 95 °C for 15 s and 60 °C for 60 s. For all qPCR experiments, three biological replicates were performed. Relative expression levels were calculated based on the 2^−ΔΔCt^ method using tubulin as a reference gene.

### 4.7. Sequence Deposition

The raw transcriptome reads reported here have been deposited in the NCBI Short Read Archive under the Accession Nos. SAMN08289417 (SL1), SAMN08289418 (SL2), SAMN08289419 (YL1), and SAMN08289420 (YL2).

## 5. Conclusions

In this study, we demonstrated senescing leaves in *L. macranthoides* can accumulate very large amounts of luteoloside, which was extremely higher than those in other leaf samples and organs including stems and flowers. Transcriptome analysis of senescing leaves and young leaves screened a subset of candidate genes associated with luteoloside biosynthesis. The elevated mRNA levels of twenty-four unigenes including *PAL2*, *C4H2*, thirteen *4CLs*, *CHS2*, six *F3′Hs* and two *UFGTs* were considered to contribute to luteoloside accumulation in senescing leaves with respect to young leaves. Thus, we hypothesized that luteoloside may be preferentially biosynthesized using the following route: naringenin-eriodictyol-luteolin. Furthermore, several unigenes encoding MYB, bHLH and WD40 TFs, such as *MYB12*, *MYB75*, *bHLH113* and *TTG1*, involved in flavonoid biosynthesis, were coexpressed with luteoloside biosynthetic unigenes in our dataset. Their roles in regulating luteoloside biosynthesis in *L. macranthoides* will be characterized via genetic and biochemical approaches in vitro and in vivo in our future work.

## Figures and Tables

**Table 1:** Statistical analysis of de novo assembly of *L. macranthoides* unigenes.

Samples	Total Number	Total Length (nt)	Mean Length (nt)	N50	Distinct Clusters	Distinct Singletons
SL-Trinity	260,079	202,070,131	777	1238	-	-
YL-Trinity	173,214	165,117,523	953	1607	-	-
SL-Unigene	183,667	165,907,666	903	1501	57,995	125,672
YL-Unigene	122,824	138,943,927	1131	1859	48,507	74,317
All-Unigene	158,842	171,974,902	1083	1743	57,337	101,505

SL-Unigene and YL-Unigene represent unigenes generated after assembling and processing the clean reads from senescing leaves (SL) and young leaves (YL) libraries, respectively. All-Unigene indicates the non-redundant unigenes generated via assembling the total clean reads from SL and YL libraries.

**Table 2:** DEGs involved in luteoloside biosynthesis during leaf senescence in *Lonicera macranthoides*.

EC Number	Accession No.	Reads Mean YL	Reads Mean SL	log2Ratio (SL/YL)	Annotation
EC:4.3.1.24	Unigene109136	0.5	66	7.44	*Solanum tuberosum PAL2*
	Unigene27450	2273.5	394.5	−2.21	*Lonicera japonica PAL3*
	Unigene29003	904	89.5	−3.01	*Lonicera japonica PAL2*
EC:1.14.13.11	CL11118.Contig2	145	1117	3.3	*Lonicera japonica C4H*
EC:6.2.1.12	CL15146.Contig1	0	40	Inf	*Arabidopsis thaliana 4CLL7*
	CL7954.Contig3	1612	138	−3.22	*Arabidopsis thaliana 4CLL10*
	Unigene100595	0	69.5	Inf	*Petroselinum crispum 4CL2*
	Unigene141050	0	165.5	Inf	*Arabidopsis thaliana 4CLL10*
	Unigene26593	1950	4922.5	1.67	*Arabidopsis thaliana 4CLL7*
	Unigene29692	1735.5	4766	1.79	*Arabidopsis thaliana 4CLL7*
	Unigene66358	0	29.5	Inf	*Oryza sativa 4CL5*
	Unigene73587	0.5	155	8.67	*Petroselinum crispum 4CL2*
	Unigene75066	0	49	Inf	*Arabidopsis thaliana 4CL4*
	Unigene80421	0	49	Inf	*Nicotiana tabacum 4CL2*
	Unigene81606	0	124	Inf	*Arabidopsis thaliana 4CLL10*
	Unigene82586	0.5	154	8.67	*Arabidopsis thaliana 4CLL1*
	Unigene84369	0	34.5	Inf	*Arabidopsis thaliana 4CLL10*
	Unigene99638	0	181.5	Inf	*Arabidopsis thaliana 4CLL7*
EC:2.3.1.74	CL11967.Contig1	5886	28.5	−7.34	*Lonicera japonica CHS*
	CL19869.Contig1	0.5	59	7.273	*Hordeum vulgare CHS2*
	Unigene12731	236	7	−4.74	*Lonicera macranthoides CHS*
	Unigene1352	5294	15.5	−8.06	*Lonicera japonica CHS*
	Unigene23683	31,797	852.5	−4.89	*Lonicera hypoglauca CHS2*
	Unigene23684	12,441.5	400	−4.63	*Lonicera japonica CHS2*
	Unigene40151	416	1	−8.35	*Lonicera japonica CHS*
	Unigene69273	1291	35	−4.88	*Lonicera japonica CHS2*
EC:5.5.1.6	CL11269.Contig3	1217.5	110.5	−3.13	*Lonicera japonica CHI2*
	CL16735.Contig2	4566	377.5	−3.27	*Lonicera japonica CHI1*
EC:1.14.11.22	Unigene2335	6778	781	−2.77	*Lonicera macranthoidesFNS II*
EC:1.14.13.21	CL11828.Contig1	1840	26,424.5	4.2	*Petunia hybrida F3′H*
	CL11828.Contig2	13.5	196.5	4.25	*Arabidopsis thaliana F3′H*
	CL7653.Contig1	0	31.5	Inf	*Solanum melongena F3′H*
	Unigene102655	0	56	Inf	*Catharanthus roseus F3′H*
	Unigene3751	799.5	21	−4.9	*Petunia hybrida F3′H*
	Unigene65437	0	44	Inf	*Arabidopsis thaliana F3′H*
	Unigene76746	0	24	Inf	*Zea mays F3′H*
	Unigene18958	38,738	1897	−4	*Petunia hybrida F3′H*
EC:2.4.1.81	CL5848.Contig3	1370	161	−2.8	*Fragaria ananassa UFGT6*
	CL8885.Contig1	1477	37.5	−5	*Fragaria ananassa UFGT6*
	CL8885.Contig2	1725	27	−5.7	*Fragaria ananassa UFGT6*
	CL8885.Contig3	707	3	−7.6	*Manihot esculenta GT2*
	Unigene2918	12	279.5	4.82	*Fragaria ananassa UFGT6*
	Unigene4567	208	3	−5.8	*Fragaria ananassa UFGT7*
	Unigene97915	5.5	114	4.69	*Fragaria ananassa UFGT6*

“Inf” and “−Inf” indicate that the Log2Ratio of SL to YL is infinity and negetive infinity, respectively.

**Table 3:** DEGs involved in MYB and bHLH family during leaf senescence.

Accession No.	Reads Mean YL	Reads Mean SL	log2Ratio (SL/YL)	Annotation
MYB				
CL11844.Contig2	363.5	35	−3.04	*Paeonia suffruticosa MYB1*
CL13949.Contig1	147.5	1.5	−6.27	*Vitis vinifera AS1*
CL13949.Contig2	88	0.5	−7.2	*Vitis vinifera AS1*
CL13949.Contig3	466	10	−5.22	*Vitis vinifera AS1*
CL14249.Contig3	267.5	1414	2.70	*Gossypium hirsutum GhMYB9*
CL14844.Contig2	39.5	0	−Inf	*Vitis vinifera MYB39*
CL15118.Contig2	3419.5	8646.5	1.67	*Vitis vinifera MYB1R1*
CL20138.Contig1	205.5	19	−3.11	*Arabidopsis thaliana TT2*
CL20138.Contig2	700.5	42	−3.75	*Arabidopsis thaliana MYB5*
CL2957.Contig1	0	163.5	Inf	*Solanum tuberosum MYB1R1*
CL3616.Contig1	2.5	258.5	7.05	*Arabidopsis thaliana MYB48*
CL3616.Contig3	35	1308.5	5.51	*Arabidopsis thaliana MYB48*
CL3616.Contig5	10	197.5	4.58	*Arabidopsis thaliana MYB48*
CL4609.Contig1	124.5	528.5	2.43	*Theobroma cacao myb2*
CL5870.Contig1	483.5	1731.5	2.21	*Vitis vinifera MYB75*
CL5870.Contig2	191	711.5	2.27	*Vitis vinifera MYB75*
CL6351.Contig2	120	9	−3.43	*Theobroma cacao Myb 106*
CL6351.Contig3	229.5	8.5	−4.4	*Theobroma cacao Myb 106*
CL8018.Contig2	5	162	5.37	*Antirrhinum majus MYB340*
CL8018.Contig4	232	6696	5.18	*Antirrhinum majus MYB305*
Unigene20238	3419	8733.5	1.71	*Epimedium sagittatum MYB13*
Unigene23056	86	360	2.42	*Petunia hybrida ODO1*
Unigene32379	1639	358.5	−1.88	*Vitis vinifera MYB44*
Unigene36582	901.5	0.5	−10.4	*Arabidopsis thaliana MYB12*
Unigene37949	59.5	0.5	−6.48	*Arabidopsis thaliana MYB46*
Unigene42805	45.5	0.5	−6.12	*Vitis vinifera MYB6*
Unigene43758	138	4	−4.85	*Vitis vinifera myb306*
Unigene47067	63.5	0	-Inf	*Solanum lycopersicum MYB46*
Unigene52460	132	0	−Inf	*Arabidopsis thaliana MYB12*
Unigene70407	1.5	61	5.68	*Oryza sativa MYB4*
bHLH				
CL10785.Contig3	243.5	0.5	−8.66	*Arabidopsis thaliana bHLH93*
CL10785.Contig6	107.5	0	−Inf	*Arabidopsis thaliana bHLH93*
CL10785.Contig7	199.5	1	−7.32	*Arabidopsis thaliana bHLH93*
CL11820.Contig21	238.5	6.5	−4.85	*Arabidopsis thaliana bHLH4*
CL11820.Contig6	119	4.5	−4.38	*Arabidopsis thaliana bHLH4*
CL12435.Contig2	327	37	−2.81	*Arabidopsis thaliana bHLH74*
CL18193.Contig2	97	0	−Inf	*Arabidopsis thaliana bHLH25*
CL2495.Contig10	37.5	317	3.42	*Arabidopsis thaliana bHLH78*
CL2495.Contig3	82	904.5	3.79	*Arabidopsis thaliana bHLH78*
CL2495.Contig7	25.5	218	3.42	*Arabidopsis thaliana bHLH78*
CL2495.Contig9	10.5	111	3.72	*Arabidopsis thaliana bHLH78*
CL3128.Contig3	384.5	1947	2.7	*Arabidopsis thaliana bHLH47*
CL3403.Contig4	642	94.5	−2.42	*Arabidopsis thaliana bHLH93*
CL3655.Contig4	8.5	99.5	3.93	*Arabidopsis thaliana bHLH113*
CL4290.Contig2	245.5	870.5	2.18	*Arabidopsis thaliana bHLH143*
CL4720.Contig6	37	211.5	2.88	*Arabidopsis thaliana bHLH123*
CL661.Contig4	20.5	231	3.88	*Arabidopsis thaliana bHLH25*
CL7365.Contig10	145.5	467	2.02	*Arabidopsis thaliana bHLH66*
CL8229.Contig1	48	0	−Inf	*Arabidopsis thaliana bHLH135*
CL8229.Contig2	38.5	0	−Inf	*Arabidopsis thaliana bHLH135*
Unigene11884	22	106	2.65	*Arabidopsis thaliana bHLH113*
Unigene19084	1634	43.5	−4.9	*Arabidopsis thaliana bHLH117*
Unigene2295	412.5	53.5	−2.61	*Vitis vinifera bHLH74*
Unigene23034	81	351.5	2.47	*Arabidopsis thaliana bHLH147*
Unigene29099	650.5	112	−2.21	*Arabidopsis thaliana bHLH79*
Unigene33227	27	427	4.37	*Arabidopsis thaliana bHLH113*
Unigene37456	135.5	1	−6.77	*Arabidopsis thaliana bHLH71*
Unigene48118	139.5	1	−6.83	*Arabidopsis thaliana bHLH14*
Unigene6423	312.5	54.5	−2.2	*Arabidopsis thaliana bHLH79*
Unigene77068	0.5	49	7.02	*Arabidopsis thaliana bHLH49*

## Supplementary Materials

Supplementary materials can be found at http://www.mdpi.com/1422-0067/19/4/1012/s1. Table S1. Overview of RNA-Seq data; Table S2. Statistical results for functional annotation of transcriptome in *Lonicera macranthoides*; Table S3. Differentially expressed genes (DEGs) identified by transcriptomic approach during leaf senescence in *Lonicera macranthoides*; Table S4. DEGs involved in WD40 transcription factors during leaf senescence in *Lonicera macranthoides*; Table S5. Primer sequences used for qRT-PCR. Figure S1. HPLC chromatogram of luteoloside with detection at 360 nm; Figure S2. Length distribution of All-Unigene sequence; Figure S3. Statistical analysis results of unigene sequences against the NCBI non-redundant protein (Nr) database; Figure S4. The Pearson and Spearman correlation coefficients of gene expression data between the biological replicates. Figure S5. Identification of significantly altered KEGG pathways during leaf senescence in Lonicera macranthoides. The left Y-axis indicates the top 20 enrichment KEGG pathways between young and senescing leaves. The different dot sizes indicate the number of DEGs corresponding to each KEGG pathway; Figure S6. Phylogenetic analysis of putative F3′Hs and UFGTs in Lonicera macranthoides. The phylogenetic tree was constructed using MEGA software (version 5.1) based on Neighbor-Joining method. Values above the branches are bootstrap percentages (1000 replicates). (A). F3H Gene/Protein ID was as follows. Nicotiana tabacum F3H, XP_016434126.1; Sesamum indicum F3H, XP_011095827.1; Solanum tuberosum cytochrome P450 71A1-like, XP_006363444.1; Solanum tuberosum F3H, XP_006355423.1; Ziziphus jujuba F3H, XP_015889029.1; Solanum lycopersicum F3H, XP_004250647.1; Nicotiana tabacum CYP92B2v3, ABC69387.1; Arabidopsis lyrata subsp. lyrata F3H, XP_020878236.1; *Arabidopsis thaliana* CYP71B37, OAP05943.1; *Arabidopsis thaliana* CYP78A10, OAP13456.1; Oryza sativa F3H, CYP75B3(Park et al.,2016). (B). UFGT Gene/Protein ID was as follows. Prunus mume UGT71A16, XP_008229642.1; Fragaria vesca UF3GT6, XP_004303955.1; Panax ginseng UGTpg19, AKA44592.1; Panax ginseng UGTPg42, AKA44601.1; Catharanthus roseus UGT, BAD29721.1; Eucommia ulmoides glucosyltransferase, AHX74090.1; Solanum pennellii UA3GT2, XP_015081102.1; *Arabidopsis thaliana* F7GT, AAL90934.1; Scutellaria baicalensis UF7GT, BAA83484.1; Nicotiana tabacum UGT71E, XP_016498099.1; Ziziphus jujube UA3GT2, XP_015880714.1; Solanum lycopersicum UF3GT6, XP_004243651.1; Nicotiana tomentosiformis UF3GT6, XP_009587669.1; Citrus sinensis UA3GT2, XP_006481379.1; Capsicum annuum UF3GT6, XP_016547987.1; *Arabidopsis thaliana* F3G7GT, Q9ZQ95.1; *Arabidopsis thaliana* F5GT, AAM91686.1; Glycyrrhiza echinata IF7GT, BAC78438.1. The putative F3H and UFGT proteins were labeled in black dots.
